# Severe Acute Respiratory Syndrome Coronavirus 2 Spike Protein Based Novel Epitopes Induce Potent Immune Responses *in vivo* and Inhibit Viral Replication *in vitro*

**DOI:** 10.3389/fimmu.2021.613045

**Published:** 2021-03-26

**Authors:** Preeti Vishwakarma, Naveen Yadav, Zaigham Abbas Rizvi, Naseem Ahmed Khan, Adarsh Kumar Chiranjivi, Shailendra Mani, Manish Bansal, Prabhanjan Dwivedi, Tripti Shrivastava, Rajesh Kumar, Amit Awasthi, Shubbir Ahmed, Sweety Samal

**Affiliations:** Translational Health Science & Technology Institute, National Capital Region (NCR) Biotech Science Cluster, Faridabad, India

**Keywords:** spike (S) glycoprotein, peptide, vaccine, antiviral activity, immune response

## Abstract

Severe acute respiratory syndrome coronavirus 2 (SARS-CoV-2) initiates infection by attachment of the surface-exposed spike glycoprotein to the host cell receptors. The spike glycoprotein (S) is a promising target for inducing immune responses and providing protection; thus the ongoing efforts for the SARS-CoV-2 vaccine and therapeutic developments are mostly spiraling around S glycoprotein. The matured functional spike glycoprotein is presented on the virion surface as trimers, which contain two subunits, such as S1 (virus attachment) and S2 (virus fusion). The S1 subunit harbors the N-terminal domain (NTD) and the receptor-binding domain (RBD). The RBD is responsible for binding to host-cellular receptor angiotensin-converting enzyme 2 (ACE2). The NTD and RBD of S1, and the S2 of S glycoprotein are the major structural moieties to design and develop spike-based vaccine candidates and therapeutics. Here, we have identified three novel epitopes (20-amino acid peptides) in the regions NTD, RBD, and S2 domains, respectively, by structural and immunoinformatic analysis. We have shown as a proof of principle in the murine model, the potential role of these novel epitopes in-inducing humoral and cellular immune responses. Further analysis has shown that RBD and S2 directed epitopes were able to efficiently inhibit the replication of SARS-CoV-2 wild-type virus *in vitro* suggesting their role as virus entry inhibitors. Structural analysis revealed that S2-epitope is a part of the heptad repeat 2 (HR2) domain which might have plausible inhibitory effects on virus fusion. Taken together, this study discovered novel epitopes that might have important implications in the development of potential SARS-CoV-2 spike-based vaccine and therapeutics.

## Introduction

The global pandemic caused by severe acute respiratory syndrome coronavirus 2 (SARS-CoV-2) has affected more than 20 million people worldwide, so far, and the spread of new infections continues to rise (1). The SARS-CoV-2 belongs to the Coronavirus family and is grouped under genus beta-corona viruses and the virus closely resembles other important Coronaviruses, including SARS and the Middle East respiratory syndrome (MERS) (2, 3). SARS-CoV-2 is an enveloped, positive-sense, single-stranded RNA virus that initiates the infection by binding to the host-cell receptor human angiotensin-converting enzyme 2 (hACE2) which is abundantly present in the respiratory tract (4). The viral genome consists of four structural proteins, such as spike (S) glycoprotein, membrane (M), nucleocapsid (N) and envelope (E) proteins, and 16 non-structural proteins (5). As per the drafted landscape of WHO for COVID-19 vaccine candidates, one-third of the vaccine development strategies are based on S glycoprotein, the major surface glycoprotein that is protruded out on the virion surface (6, 7). A suitable viral spike protein vaccine candidate should either mimic the native-like structure to induce naïve B-cell repertoire for neutralizing antibodies or should incorporate highly immunogenic viral sequences that will have the advantage to bind at high affinity to MHC class I/II molecule, thus stimulating host immune responses.

The SARS-CoV-2 S protein is a type I transmembrane glycoprotein which remains in a metastable conformation and three homo-dimeric complexes assemble to form trimers that are exposed on the virion surface (8). The S glycoprotein initiates the infection by attachment to hACE2 receptor present on the host cell surface, thus stimulating conformational changes for the fusion of the viral-host cell membrane (6). Thus, the SARS-CoV-2 S protein is the major target for eliciting neutralizing antibodies and to confer protection; however, an ideal vaccine candidate should be the one that will be able to induce both humoral and T-cell responses. The T-cell responses have great importance in the clearance of respiratory viruses. The S protein is a 180 kDa glycoprotein and contains three major domains; the ectodomain (S1 and S2 domains), transmembrane domain, and intracellular cytoplasmic domain. The S1 domain consists of N-terminal domain (NTD) and receptor-binding domain (RBD) that binds to the host cell receptor hACE2, and initiates attachment. The C-terminal domain (CTD); i.e., the S2 domain which contains the highly conserved fusion peptide and heptad repeat (HR) domains, mediates fusion process and facilitates virus entry into the host cell (9). The fusion process requires activated S protein which is achieved in the host secretory pathway by cleavage of the S protein by host cell proteases, and the cleavage site is present at the S1/S2 site and the S2′ site (10). Other than the similarities with SARS-CoV from the coronavirus family, the spike protein resembles in its structural organization and functions to other class 1 viral envelope and fusion proteins, such as HIV-1 envelop gp160, HCV, chikungunya, HA protein from influenza virus, and Ebola GP protein (11). The dynamic conformational flexibility of these class 1 viral proteins facilitates extraordinary attachment and fusion process between viral and host cell membranes and responsible for inducing both B- and T- cell immune arms. Hence, the structural proteins exposed on virion surfaces are excellent targets for vaccine and therapeutic development. Nevertheless, to exploit the spike protein up to its full potential to be used as a vaccine or therapeutics, it is utmost necessary to have a comprehensive understanding of the monomeric and trimeric structural and functional characteristics of spike protein and its different epitopes.

Based on the earlier knowledge on the efforts of development of SARS-CoV vaccine and target product profiling and recent studies on SARS-CoV-2 vaccine development, S protein-based subunit vaccines have shown to induce immune responses (12–15). The whole trimeric S protein might be a potential vaccine candidate; however, in SARS it has been seen that in some cases, the full-length S protein induces enhanced infectivity and infiltration of eosinophils which might be due to antibody-dependent enhancement (ADE) effect (16–18). Another important aspect is that as the virus is adapting to the host and evolving, the mutations in the SARS-CoV-2 genome might act as an important bottleneck in the vaccine development (19, 20), whereas, the usage of the receptor-binding domain (RBD) alone could avoid ADE effect and can elicit the desired neutralizing responses. In SARS-CoV-2, it is too early to speculate which of these strategies will work and would be beneficial. Hence, it is important to test potential domains or epitopes of S protein and understand their roles in either inducing immune responses or in viral attachment and fusion process.

In this study, we have identified novel epitopes and synthesized 20 amino acid peptides from the S protein of SARS-CoV-2 from three major domains i.e., NTD (S1 domain), RBD, and CTD (S2 domain) by selecting minimal viral epitopes. These epitopes showed promising antigenicity using structural and immunoinformatic analysis, and we have further characterized their immunogenicity and as anti-viral entry inhibitors both *in vivo* and *in vitro*, respectively. Single domain peptides with minimal length have advantages as these are easy to produce, cost-effective, less complex, and easy to remove the unwanted non-neutralizing epitopes or domains. In addition, epitopes showing promising results could be stitched together to form multiepitope protein-based immunogens (21) or could be formulated into nanoparticles (22). In addition the epitopes have shown to induce neutralizing responses in SARS-CoV (23, 24). The immunogenicity of these peptides was evaluated in 7–8 weeks old female Balb/c mouse by a prime-boost approach, given either as a single dose or in a combination of three peptides. The results indicate all three peptides are highly immunogenic and able to induce both antigen-specific soluble spike protein and RBD protein-specific antibody responses and in mixed or cocktail preparation of peptides; the antibody responses were found to be biased toward S2 and RBD domain. We have further analyzed the functional and neutralizing responses of the sera using SARS-CoV-2 wild type viruses, antigen-specific T-cell responses, and the ability of these peptides to block SARS-CoV-2 virus entry before and after the virus attachment. Altogether, the results demonstrate valuable insights of these novel epitopes derived from SARS-CoV-2 spike protein and describe their potential application in the designing of next-generation improved vaccine candidates or as anti-viral “gate-keepers”.

## Results

### Immunoinformatic and Structural Analysis of S Protein, Peptide Design, and Synthesis

**Figure 1:**
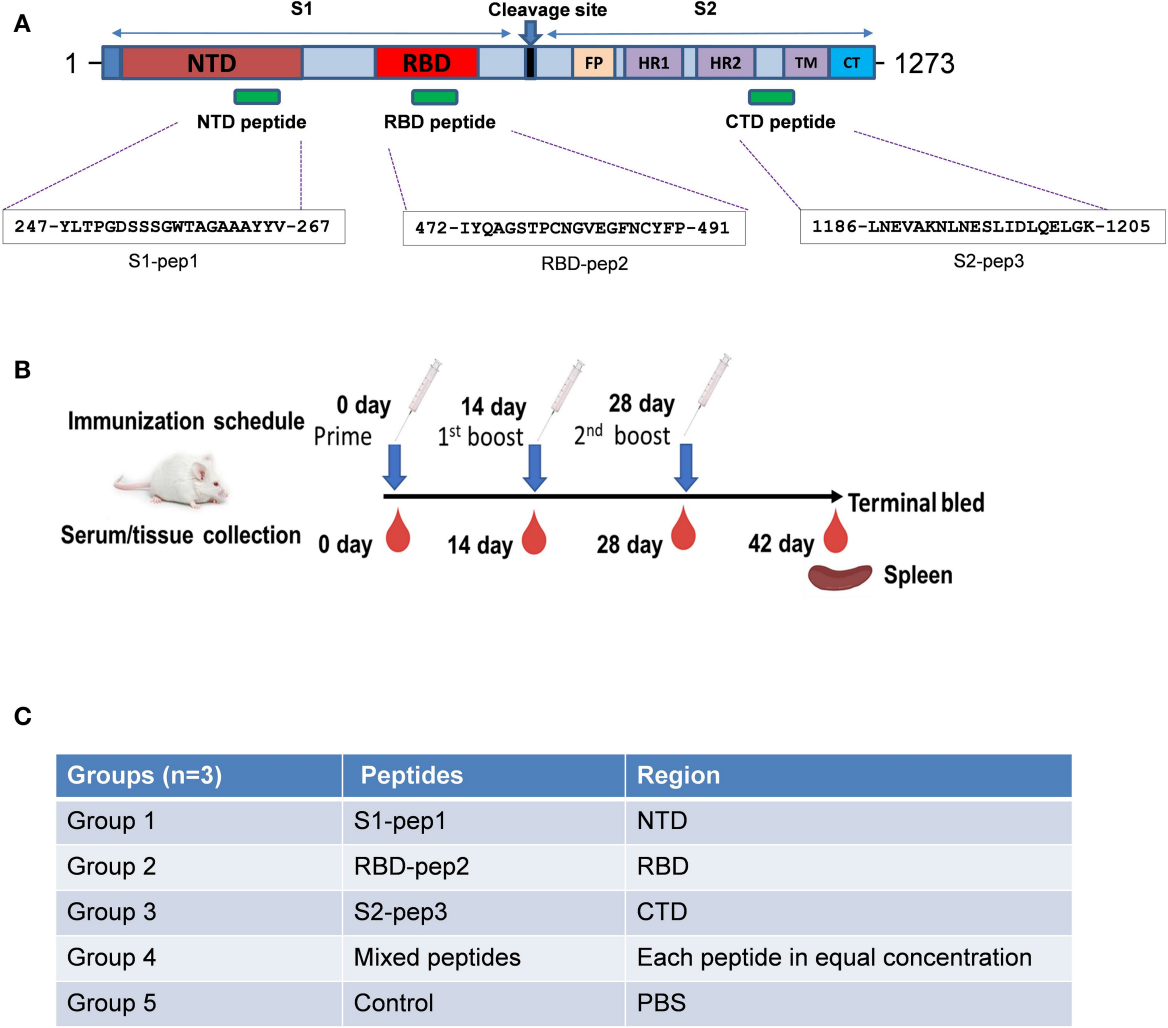
Schematic of SARS-CoV-2 spike protein and immunization schedule. **(A)** Full-length spike protein depicting sequence location of S1-pep1, RBD-pep2, and S2-pep3. **(B)** Immunization scheme and sample collection schedule in BALB/c mice. **(C)** Immunization strategy in different groups.

The SARS-CoV-2 spike protein is the major immunogenic candidate present on the virion surface and protective neutralizing antibodies targeting the spike protein are present in the samples from convalescent patients. Literature suggests that for SARS coronaviruses, neutralizing antibodies are elicited against different domains of the spike protein (23, 25). Here, we analyzed the S glycoprotein of SARS-CoV-2 (Wuhan-Hu-1 strain, GenBank accession ID: MN908947.3) and identified immunogenic peptides from three different regions: from the S1-NTD named as S1-pep1, from the RBD region named as RBD-pep2, and from the S2 region named as S2-pep3 (Figure 1A and Supplementary Figure 1). The peptide selected from the NTD corresponds to a previously reported peptide from SARS-CoV NTD (23). The NTD peptide was evaluated for B-cell linear epitope prediction and showed favorable values for the predicted antigenicity. The peptide selected from the RBD is a part of the receptor-binding motif (RBM). The corresponding peptide from the SARS-CoV is known to bind to neutralizing antibodies, such as F26G18 and F26G8 (24). Linear B cell epitope prediction showed favorable predicted antigenicity for the RBD-pep2. We have further identified another peptide from the C-terminal region in the HRC1 sequence of S2 domain as described by Tripet et al. (26), who has shown that in SARS-CoV, this HRC1 epitope site was an effective viral inhibitor. The selected peptides were synthesized and conjugated to the keyhole limpet hemocyanin (KLH) (GenScript, NJ, USA), a carrier protein, to increase their immunogenicity. The peptide sequences are listed as shown in Figure 1A.

### Immunization of Peptides in Mice Induces High-Titer Antibody Responses

**Figure 2:**
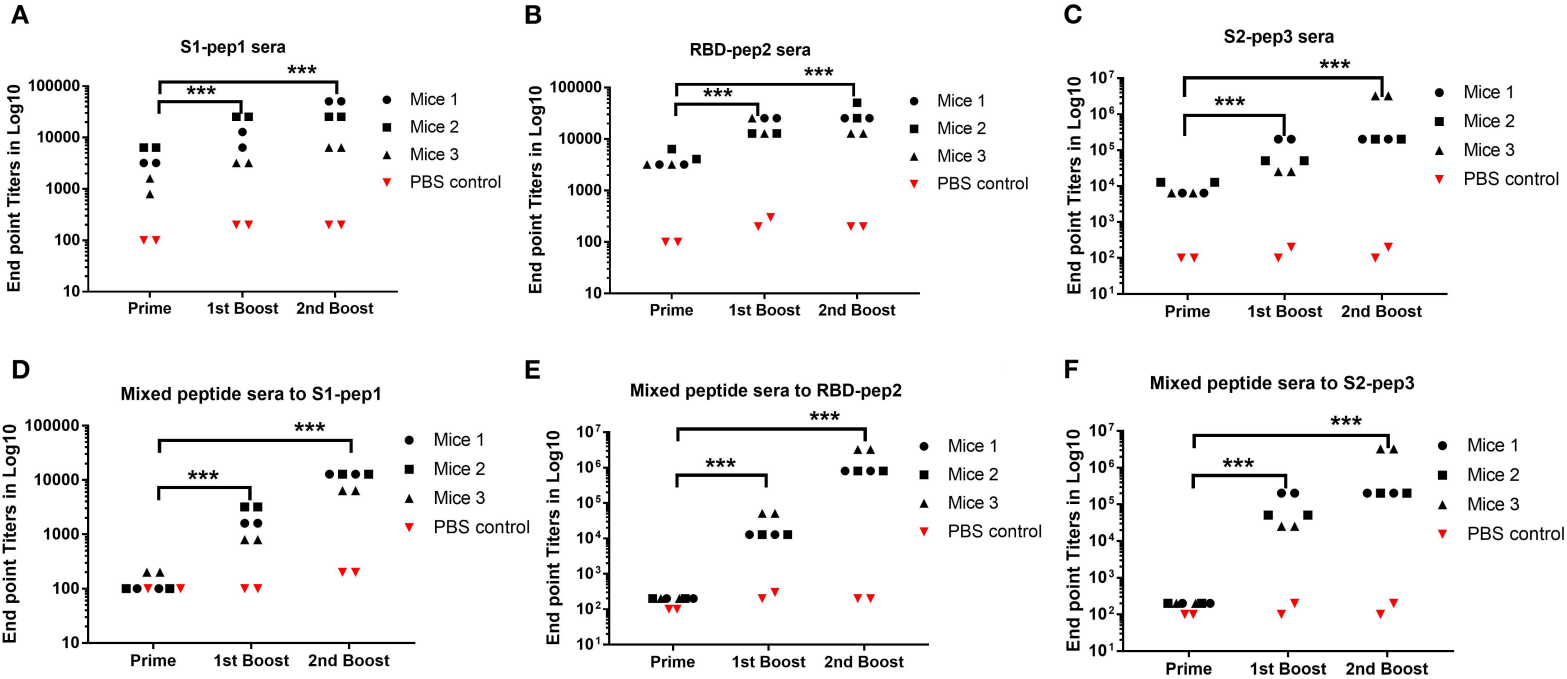
Humoral responses to peptide immunization. Serum whole IgG responses as measured by ELISA and expressed as endpoint antibody titer (Mice no. 1–12). **(A)** Binding antibody titers of immunized Group 1 to S1-pep1. **(B)** Binding antibody titers of immunized Group 2 to RBD-pep2. **(C)** Binding antibody titers of immunized Group 3 to S2-pep 3, sera from priming, sera from the first boost, and sera from the second boost. **(D)** Binding antibody titers from the first boost of immunized Group 4 mixed peptide sera to S1-pep1. **(E)** Binding antibody titers from the first boost of immunized Group 4 mixed peptide sera to RBD-pep2. **(F)** Binding antibody titers from immunized Group 4 to S2-pep3. The plates were coated with respective peptides at a concentration of 2 μg/ml. Values plotted are the mean endpoint titers in duplicate. Each dot represents a single mean value of three repeated experiments. Statistical significance was determined using the one-way ANOVA test (*p* < 0.05), where ***p < 0.001 (one-way ANOVA).

To determine the immunogenicity of these peptides, we immunized 7–8-week-old BALB/c mice in a homologous prime-boost regimen (Figures 1B,C) as described in “Materials and Methods.” The whole IgG responses of the immunized sera from four experimental groups and one control group were measured against S1-pep1, RBD-pep2, S2-pep3, and mixed peptide group, respectively by ELISA (Figure 2). After priming the immunized sera from all the groups, we were able to observe elicited whole IgG titers (Figures 2A–C). After the first and second boosting, the whole IgG antibody responses were increased (Figures 2A–C), whereas, the highest IgG titer was seen in S2-pep3 group followed by RBD-pep2 and S1-pep1 immunized groups. The immunized sera from the mixed peptides (Group 4) did not show any reactivity toward respective peptides after priming (Figures 2D–F). However, after the first and second boost, the sera from the immunized group of mixed peptides showed a significant increase in IgG responses, and the highest responses were directed toward S2-pep3 (Figure 2F). The control group with PBS did not show reactivity to any of the peptides.

Next, we investigated peptide-specific IgG subclass switching in sera from the second boost immunization to determine Th1/Th2 polarization (Supplementary Figure 2). IgG1 subclass was found to be dominated in the sera from the immunized mice with all the three peptides (Supplementary Figure 2A), whereas, no titer was detected against IgG2c subclass (Supplementary Figure 2D). In the S1-pep1 immunized sera, there was no significant titer against IgG2a and 2b (Supplementary Figures 2B,C, left panel). In the RBD-pep2 immunized sera, high IgG2b titers were seen (Supplementary Figure 2C, middle panel), followed by IgG2a responses (Supplementary Figure 2B, middle panel). Immunization with S2-pep3 also induces significantly higher titers to IgG2b (Supplementary Figure 2C, right panel). In contrast, in the immunized sera of mixed peptides, the IgG1 titers are found to be the highest against S2-pep3 followed by RBD-pep2 and S1-pep1 (Supplementary Figure 2E). In addition, the mixed immunized sera showed the highest IgG2a and moderate IgG2b titers to S2-pep3 (Supplementary Figure 2F, left and middle panels) followed by reactivity to RBD-pep2 (Supplementary Figure 2G, left and middle panels); however, no reactivity was shown to IgG2c (Supplementary Figures 2F,G, right panel). In addition, the mixed immunized mice sera showed a very low titer of IgG2a, 2b, and 2c against S1-pep1 (data not shown).

### Peptide Prime-Boost Immunization Efficiently Mounted Antigen-Specific CD8+ T Cell Responses

**Figure 3:**
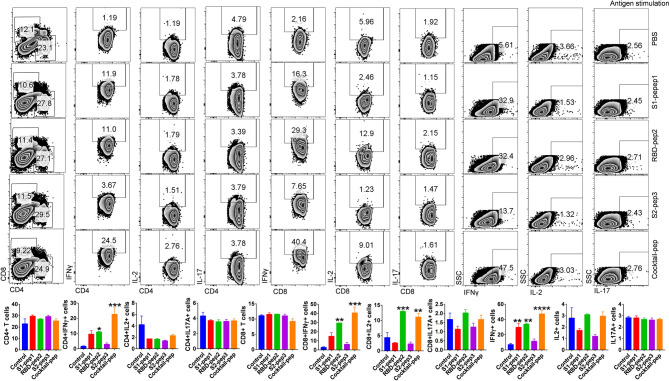
Characterization of T-cell immune responses against SARS-CoV-2 antigens in mice. T-cell immune response against SARS-CoV-2 peptide antigens was studied in the spleen of immunized mice at 42 days post-immunization (p.i.). *In vitro* antigen-stimulated splenocytes were then used for intracellular cytokine staining for IFNγ, IL2, and IL17 cytokines after CD4 and CD8 surface staining. Stained cells were then acquired on BD FACSCanto II (BD Biosciences, CA, USA) and analyzed on FlowJo software (Tree Star, Inc., OR, USA). The top panel shows representative dot plots indicating mean percent positive values for various T-cell populations in the stimulated spleen. Bar graph plotted for percent positive ± standard errors of the mean (SEMs) for each group are shown (Bottom), where*p < 0.05, **p < 0.01, ***p < 0.001, and ****p < 0.0001 (one-way ANOVA).

T-cell responses to S1-pep1, RBD-pep2, and S2-pep3 and mixed peptide preparation were characterized in the mice immunized with respective peptides. For this purpose, spleens were isolated from each of the above-immunized groups and stimulated *in vitro* in the presence of PMA + Ionomycin or respective peptide antigen and compared with the mock control group. Characterization of various T-cell populations was then carried out based on the presence of CD4, CD8 surface markers, and cytokines. In peptide stimulated groups, both the T helper as well as T cytotoxic cells showed ~10-fold upregulation in IFN-γ (Interferon-gamma) production in the mixed peptide immunized mice followed by RBD-pep2 group which showed ~6-fold upregulation as compared to the control group (Figure 3). Similar significant upregulation of total IFN-γ production was also observed in the presence of mixed peptide and RBD-pep2, indicating a robust IFN-γ mediated anti-viral response in the presence of these peptides (Figure 3). Consistent with the antigen-specific stimulation, PMA + Ionomycin stimulation also resulted in significant IFN-γ production in the mixed-peptide group and RBD-pep2 immunized group (Supplementary Figure 3). Interestingly, the upregulated IFN-γ production was also observed in the case of S1-pep1 group in both antigen-specific and PMA+Ionomycin stimulation. However, no significant changes were observed either in IL2 or IL17A production suggesting that our immunization strategy particularly with mixed peptide, RBD-pep2, and S1-pep1 induces a strong IFN-γ production which may be driven by Th1 axis.

### Functional and Biological Characterization of SARS-CoV-2 Peptide Immunized Sera

**Figure 4:**
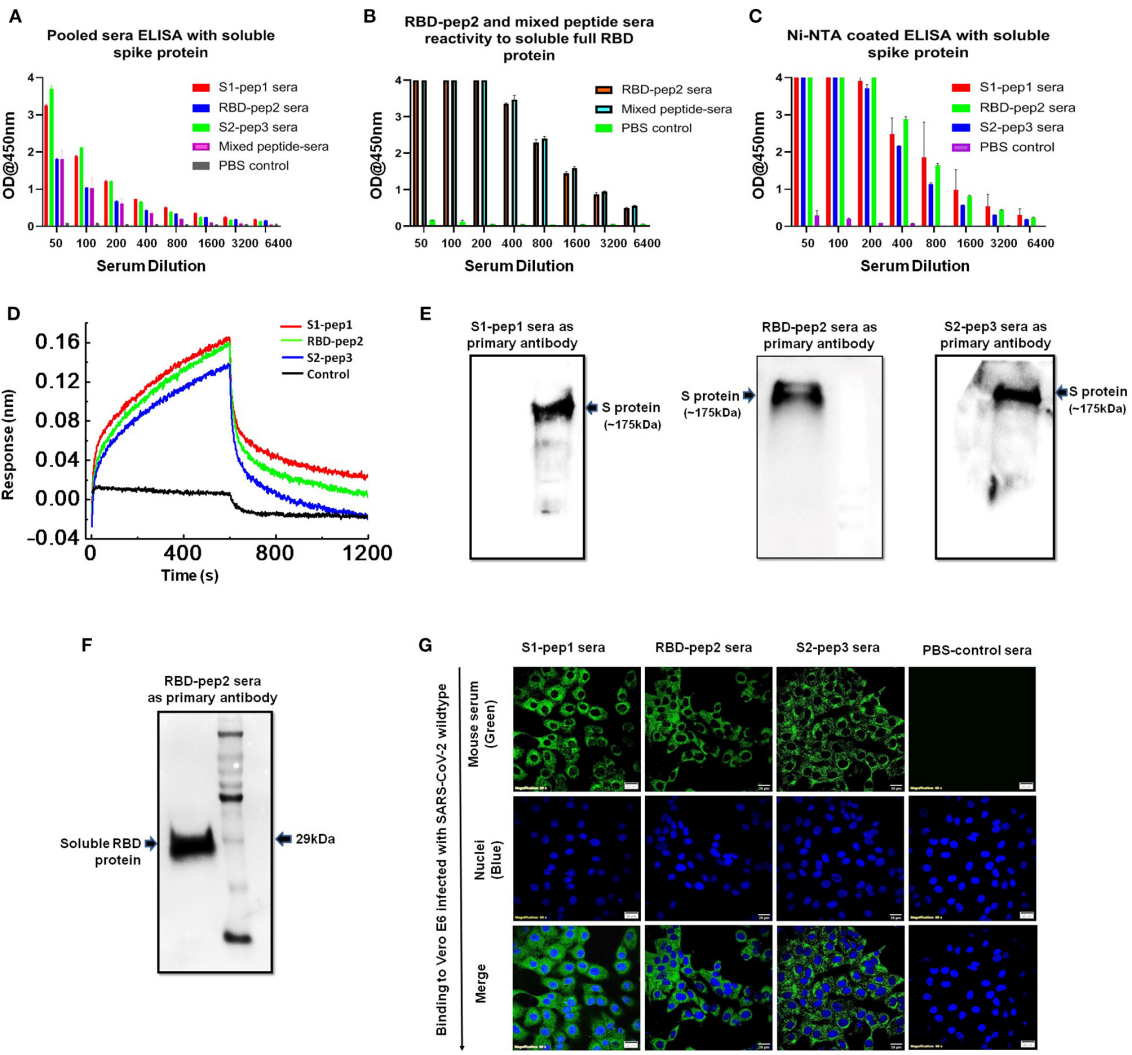
Functional characterization of immunized sera. (A) Binding antibody titers of immunized pooled sera from S1-pep1 (red), RBD-pep2 (blue), S2-pep3 (green), and mixed peptide sera (pink) to the soluble spike protein. **(B)** Binding antibody titers of immunized sera from RBD and mixed peptide-immunized pooled sera to the soluble RBD protein. **(C)** Binding titers of pooled sera to Ni-NTA HisSorb-captured spike protein ELISA. **(D)** Real-time binding of soluble spike protein with the purified IgGs from immunized mice, S1-pep1 (red), RBD-pep2 (green), and S2-pep3 (blue). **(E)** The Western blot analysis of pooled immunized sera reactivity to soluble spike protein separated on SDS-PAGE (4–12%) gels. The pooled sera from S1-pep immunized group (lane), RBD pep (middle lane), and S2-pep (right lane) have been used as the primary antibody. **(F)** The Western blot analysis of pooled immunized sera from RBD-pep2 group as the primary antibody to soluble RBD protein separated on SDS-PAGE (4–12%) gels. **(G)** Immunofluorescence detection of pooled immunized peptide sera to wild type SARS-CoV-2 virus-infected Vero E6 cells. Immunized sera as the primary antibody and probed with secondary antibody. Alexa-Fluor 488-labeled anti-mouse antibody (green) (1:100). The nuclei were stained with 4′,6-diamidino-2-phenylindole (DAPI, blue); scale bar: 20 μm and magnification 60x.

Next, we determined the soluble spike protein (in pre-fusion-stabilized conformation) and soluble RBD-specific antibody titers of the peptides immunized sera from the second boost. The highest total IgG titers against the soluble spike protein were seen against S2-pep3 immunized mice pooled sera (Figure 4A). These data corroborate with the earlier results that S2-pep3 is highly immunogenic in BALB/c mice. Pooled sera from S1-pep1 immunized mice showed comparable ELISA titers to soluble spike protein and the lowest IgG titers were seen in RBD-pep2 immunized mice pooled sera. However, the reactivity of high titer antibodies was seen in RBD-pep2 immunized mice pooled sera and in the mixed peptide sera to soluble RBD protein (Figure 4B). To further confirm whether the induced antibodies recognize the conformational spike protein, Ni-NTAHisSorb ELISA plates were used to coat spike protein, and ELISA was repeated with pooled sera from S1-pep1, RBD-pep2, and S2-pep3, respectively (Figure 4C). The pooled sera from all the three peptides showed binding to conformationally stable spike protein and interestingly, the sera showed higher binding titers to soluble spike protein in Ni-NTA coated plates as compared to the binding titer to ELISA. Next, we analyzed the binding of IgGs purified from the pooled sera of the immunized mice to assess their binding with a soluble spike in pre-fusion conformation in real-time using bio-layer interferometry (BLI). All the sera, the S1-pep1, RBD-pep2, and S2-pep3, were found to bind with the soluble spike protein; this further confirms that the immunized sera have antibodies that are specific to the spike protein of SARS-CoV-2 and bind to the soluble, pre-fusion, native-like, trimeric spike protein (Figure 4D). We further analyzed the ability of the immunized pooled sera to bind selectively to soluble spike and RBD protein by Western blot (Figures 4E,F). The sera from each immunized group were pooled and as shown in Figure 4E, sera from the S1-pep1, RBD-pep2, and S2-pep3 immunized groups efficiently detected the soluble spike protein at 1:100 dilution. Similarly, pooled sera from RBD-pep2 immunized sera detected soluble RBD protein at 1:1000 dilutions (Figure 4F). The sera from the pooled peptides were able to detect the soluble spike protein as a clear single band at an approximate molecular weight of ~175kDa, whereas, the pooled peptide sera from RBD-pep2 recognized the soluble RBD as a single band at an approximate molecular weight of ~29 kDa (27, 28). To study the ability of the sera from immunized groups to detect expressed spike protein in viruses, Vero-E6 cells were infected with wild type SARS-CoV-2, Isolate USA-WA1/2020 virus using an MOI of 10 and 24 h p.i., the binding of the pooled sera from each immunized group to viruses was determined by indirect immunofluorescence (Figure 4G). Pooled sera from all S1-pep1, RBD-pep2, and S2-pep3 immunized mice were shown to be effectively binding to spike proteins intracellularly. Taken together, all these assays show that the immunized sera from the 20-mer peptides were effectively recognized by the soluble RBD protein, the soluble spike protein stabilized in pre-fusion conformation mimicking native-like structure, and the spike protein expressed in the virus-infected cells.

### Neutralizing Responses of the Anti-peptide Sera and Anti-viral Properties of Peptides to Wild-Type SARS-CoV-2 Virus

**Figure 5:**
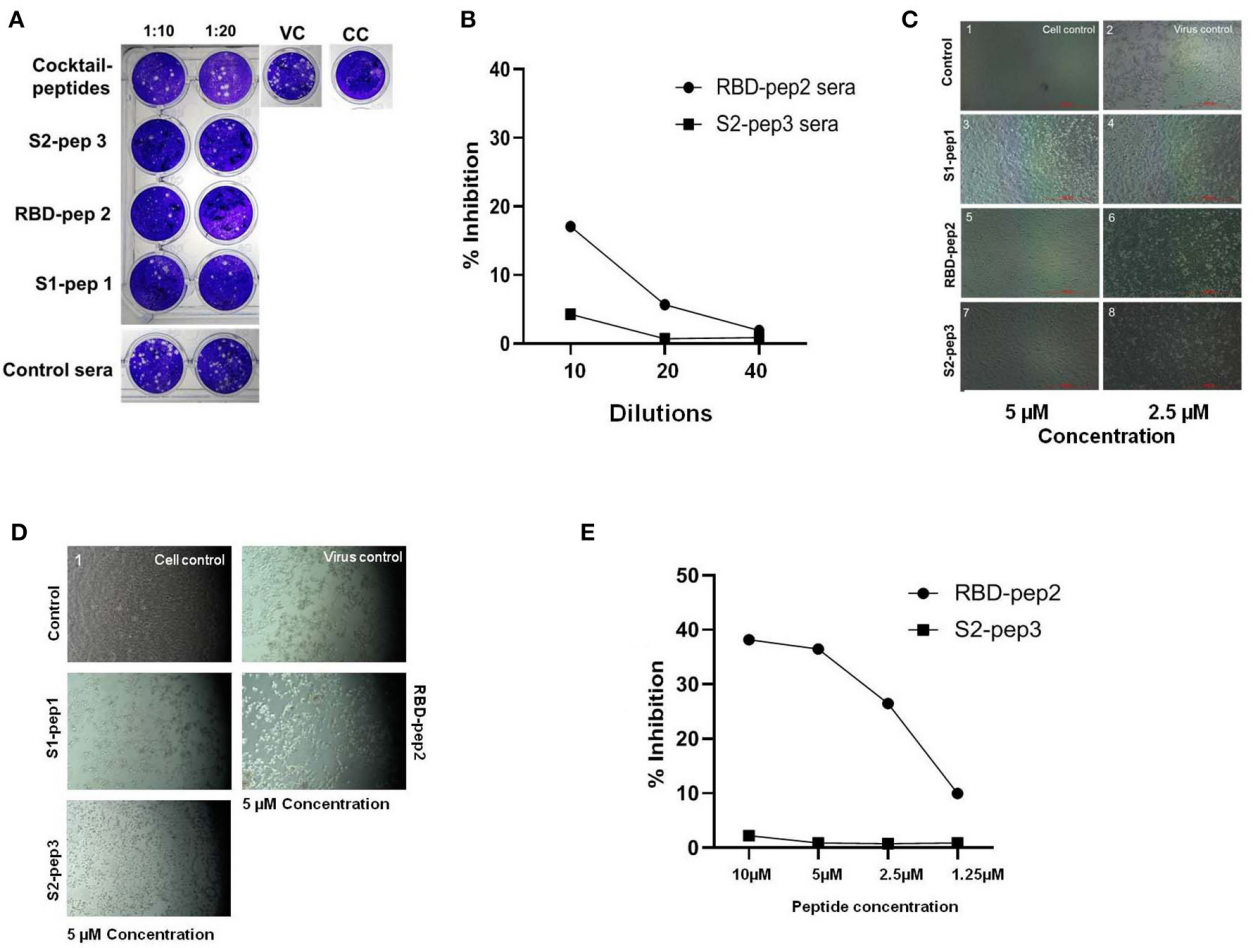
*In vitro* neutralization and anti-viral inhibition assay. **(A)** Plaque assays were performed using SARS-CoV-2 (USA-WA1/2020) isolate in Vero E6 cells. Sera were incubated with 50 PFU of viruses at 1:10 and 1:20 dilutions for 1 h at room temperature (RT) and allowed to infect the Vero E6 monolayer of cells seeded onto 24-well plates. Following 1 h of adsorption, the plate was washed with 2% Dulbecco's Modified Eagle Medium (DMEM) and overlaid with 2% carboxymethyl cellulose (CMC) prepared in DMEM. Forty-eight hour p.i., the plates were fixed with 6% formalin for 4 h at RT and stained with 1% crystal violet to visualize the plaques. VC, Virus control; CC, cell control. **(B)** The ACE2-RBD competition assay was performed in ELISA format showing percentage inhibition of SARS-CoV-2 RBD binding ACE2 in the presence of RBD-pep2 sera. **(C)** Cytopathic effect-(CPE) based viral inhibition assay with peptides. The peptides with different concentrations were incubated with 10 MOI of wild type SARS-CoV-2 virus for 30 min at RT and allowed to infect the Vero E6 monolayer of cells seeded onto 12-well plates. After 1 h of adsorption, the plate was washed and maintained in 2% DMEM growth media. Forty-eight hour p.i., the CPE were visualized in the microscope. **(D)** The CPE-based viral inhibition assay with peptides after virus attachment. **(E)** The ACE2-RBD competition ELISA with RBD-pep 2. The RBD-pep2 was incubated at different concentrations with 2 μg of soluble RBD protein for 30 min at RT. The mix was then transferred to the ELISA plate coated with soluble ACE2 and further incubated for 1 h at RT. The plate was then washed and an HRP- conjugated anti-His antibody was added and incubated for 1 h at RT; the plate was washed two times with PBST and then developed with a TMB substrate. The reaction was stopped by adding 2N H_2_SO_4_ stop solution and absorbance was measured at 450 nm.

The ability of immunized sera of peptides to neutralize the SARS-CoV-2 was evaluated by using classical virus plaque-based neutralization assay in Vero E6 cells (Figure 5A and Supplementary Figure 4A, high resolution). The pooled sera from the peptide of immunized groups after the second boost were incubated with 50 PFU of SARS-CoV-2 (USA-WA1/2020 isolate) at 1:10 and 1:20 dilutions, respectively and 48 h p.i. The number of plaques formed was visualized by crystal violet staining. As shown in Figure 5A, the pooled sera from all groups immunized with peptide showed to neutralize the SARS-CoV-2 virus as compared to the immunized sera of the control group. The sera from the mixed immunized mice group showed higher plaque counts at 10 and 20 dilutions as compared to others, suggesting least neutralization ability. We further tested the ability of the RBD-pep2 immunized sera to block ACE2-RBD interaction by using surrogate neutralization assay. As shown in Figure 5B at 1:10 dilution, the pooled sera from RBD-pep2 immunized mice from the second boost showed ~17% inhibition in binding of RBD to ACE2 in competitive ELISA. The result was in agreement with plaque reduction neutralization test (PRNT) indicating that the 20-mer RBD-pep2 harbors the neutralizing epitope. Although the neutralizing titers were low, this might be attributed to the small size or low dose of the peptides or both, and the adjuvant used. Nevertheless, the 20-mer peptides were able to induce neutralizing responses albeit in weak concentration.

Next, we examined the inhibitory effect of these synthetic peptides on the SARS-CoV-2 virus, which should prevent either virus attachment or fusion process, and thereby preventing the virus entry into the host cells. To test whether the peptides were able to inhibit the virus in the pre-fusion state before they entered into host cells, the peptides were incubated with 10 MOI of SARS-CoV-2 (USA-WA1/2020 isolate), free viruses for 30 min at concentrations of ~5 and 2.5 μm, and at 48 h p.i. The cytopathic effect (CPE) was visualized in Vero E6 cells (Figure 5C and Supplementary Figure 4B, high resolution). As compared to virus control, all the three peptides S1-pep1, RBD-pep2, and S2-pep3 showed an inhibitory effect on wild type virus, though the highest effect was seen in RBD-pep2 and S2-pep3 peptides in which at low concentration of ~2.5 μm, the formation of CPE was found to be lower as compared to the virus control. We further tested the anti-inhibitory effect of peptides after the virus attachment or entry to host cells. For this, 10 MOI of free viruses were allowed to attach to Vero E6 for 1 h, followed by washing and addition of a medium containing peptide (10 μm). When the cells were incubated at 37°C for 48 h p.i., CPE was observed. We observed similar CPE in the S1-pep1 and RBD-pep2 wells as compared to the virus control, where ~80% of the cells were dead. For the S2-pep3, the CPE was less prominent and ~50% cells were attached to the surface (Figure 5D and Supplementary Figure 4C, high resolution). In surrogate-based ACE2-RBD competition ELISA, the RBD-pep2 showed ~37% inhibition of binding to hACE2 at 10 μm peptide concentration (Figure 5E). Taken together, the results indicate that these peptides not only have the potential to generate neutralizing responses but could also act as anti-viral inhibitors. In addition, the results suggest that S2-pep3 peptide could inhibit the virus both pre- and post-entry to host cell, although it is interesting to explore further the molecular mechanism of the S2-pep3 and the interaction of the virus spike protein.

### Structural Modeling of Peptides on the Ectodomain of SARS-CoV-2

**Figure 6:**
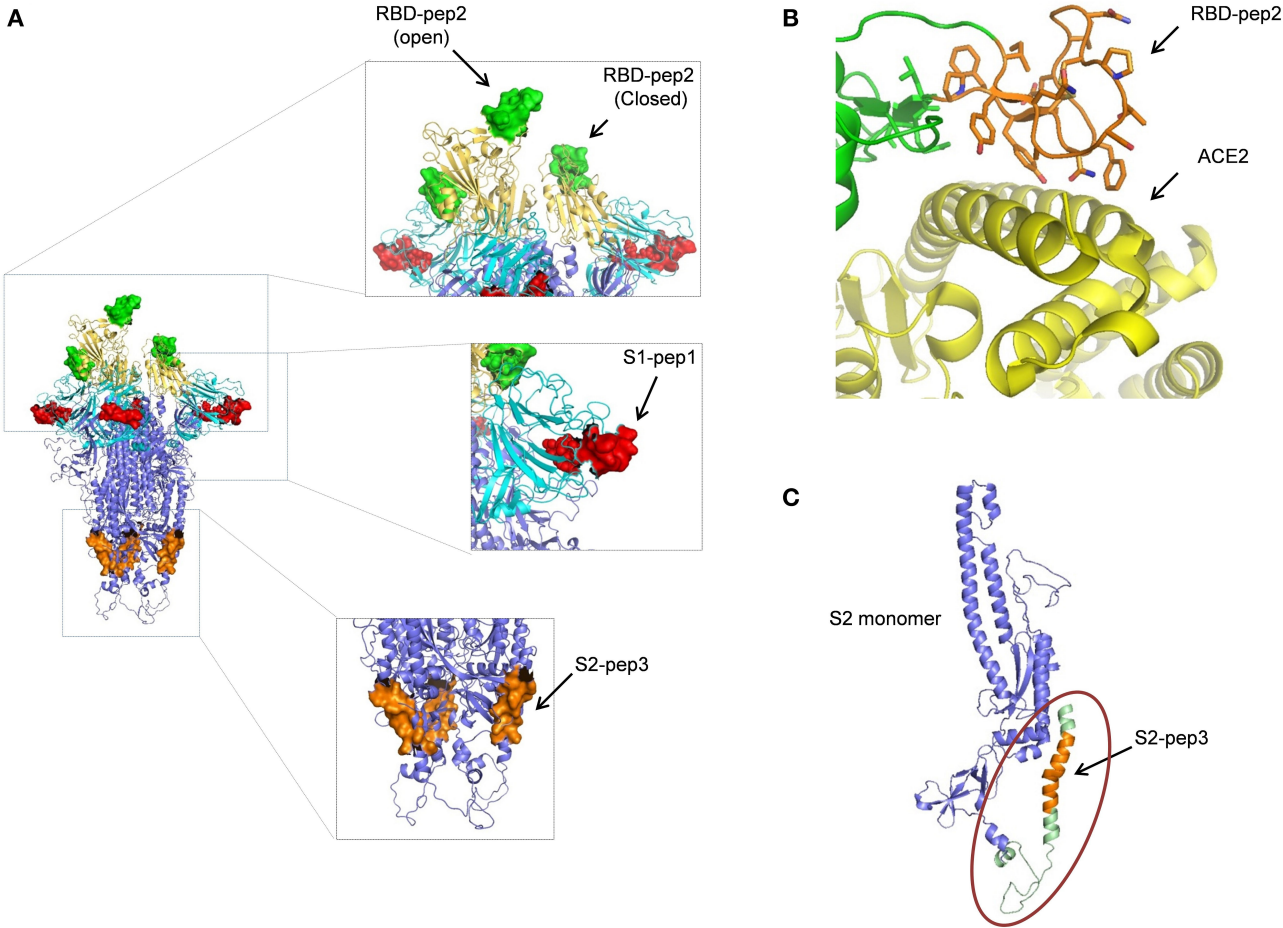
Structural modeling of peptides on SARS-CoV-2 S protein. **(A)** The position of the peptides used in this study is shown on the spike trimer. S1-pep1 is shown in red, that is part of N-terminal domain (NTD) shown in cyan, RBD-pep2 is shown in green, that is part of RBD is shown in yellow (note one RBD is in open conformation and two in closed conformation), and S2-pep3 is shown in orange, that is part of S2 domain. All the peptides are well-exposed to the trimeric spike protein. **(B)** The RBD-pep2 region in the RBD is shown in orange in the stick model. The RBD-pep2 consists of key residues that are at the interacting interface of ACE2, shown in yellow (PDB: 6M0J). **(C)** The position of the modeled S2-pep3 is shown in orange in one S2 protomer shown in blue. The structure shown in green in the circle is the C terminal of the spike protein proximal to the membrane that folds back to project upward and forms a helix. All the images were created by using the PYMOL software.

The peptides that were selected from the SARS-CoV-2 primary amino acid sequence were missing in the available structures in the protein data bank for the SARS-CoV-2 spike protein. This suggests that the regions from where the peptides selected are random and have a high b factor. To build-up the missing parts, we used SWISS-MODEL and PDB: 6VYB as a template. The structure of SARS-CoV-2 ectodomain in PDB:6VYB is in an open state, with one RBD open while the two are in the closed conformation (6). This helped us to view the state of RBD-pep2 on the SARS-CoV-2 structure both in the context of open and closed conformation (Figure 6A). The location and conformation of both, S1-pep1 and RBD-pep2 were highly exposed and easily accessible on the SARS-CoV-2 ectodomain, suggesting that antibodies for these regions will have easy accessibility on the S protein (Figure 6A). The RBD-pep2 is part of the RBM which in general, is very flexible and adopts a structure when binds to the ACE2 receptor (Figure 6B). Antibodies targeting the RBM region are neutralizing, and we hypothesized that the use of RBD-pep2 as a peptide vaccine candidate might elicit neutralizing antibodies. To build the missing region of S2-pep3 on the S protein, we used int-FOLD server (29) to model the missing C-terminal domain of S2 region. The best fit modeled S2-pep3 region shows that it forms a helix and projects upward from the proximal region of the membrane (Figure 6C). The conserved hydrophobic region that immediately precedes the trans-membrane region is also crucial for S-protein trimerization and stability. Antibodies binding to this region may destabilize the trimer formation or post-fusion conformation (30). A similar phenomenon is hypothesized in HIV envelope protein, where the region closer to the membrane induces antibodies that block the conversion from pre-fusion to post-fusion conformation during the process of cell entry (31).

## Discussion

The spike protein of SARS-CoV-2 is an important target for developing vaccines and therapeutics as it binds to cellular receptors on the host-cell membrane and mediates virus–host cell fusion. Currently, no vaccines are available for any Coronaviruses, although few promising vaccine candidates have entered into clinical trials for SARS-CoV-2 following the recent outbreak (32, 33). SARS-CoV-2 is an RNA virus with genetic diversity that results from antigenic mutations, which is a common feature of the RNA virus family (20, 34). Hence, it is valuable to evaluate and find anti-viral and neutralizing epitopes that could be used as universal targets for the designing and development of SARS-CoV-2 spike-based vaccines or as therapeutics. According to recent reports, the number of D614G mutants of SARS-CoV-2 has increased rapidly all over the world, indicating a transmission advantage and better fitness of the D614G subtype (35). This also emphasizes the importance of identifying conserved domains and epitopes on SARS-CoV-2 spike protein for broad coverage. In this study, we have selected 20-mer peptides from three important immunogenic determinants of S protein by structural and immunoinformatic analysis, and based on previous reports of SARS spike protein (24). These synthetic peptides were used to immunize BALB/c mice and the sera were characterized extensively for their antigenicity against the respective peptides, the RBD soluble protein, and the soluble pre-fusion stabilized native-like S protein. All the three peptides were found to be highly immunogenic and were able to elicit a high titer of whole IgG antibodies against the respective peptides as shown in Figure 2. However, in the immunized group of the mixed peptides, the immune responses were found to be skewed toward RBD and S2-pep3. In addition, further evaluation of immunized sera on isotype class switching demonstrates that the response is directed toward IgG1 suggesting induction of Th1 responses (Supplementary Figure 2). Interestingly, the highest humoral response was seen against S2-pep3, which is a part of the heptad repeat 2 (HR2) domain (36). In addition, our results demonstrated that the intramuscular immunization of these synthetic peptides could trigger spike-specific CD8+ T cell responses as shown with respect to the secretion of IFNγ (Figure 3). IFN-γ secretion is a hallmark of Th1 response and these results corroborated with our earlier findings of elicitation of high titers of IgG1 responses. This is crucial information on the development of the SARS-CoV-2 vaccine or therapeutics, as one of the major implications of the SARS vaccine is the induction of severe antibody-dependent inflammatory reactions (37) and Th2-directed eosinophils infiltration that results in the damage of lungs (38). The demonstration of the association of the synthetic peptides to Th1 polarizing subclasses and a balance of Th1/Th2 responses are important, as the IgG subclasses have shown to play an important role in controlling the infection of the virus. Furthermore, Th1 responses have an additional advantage for eliciting CD8^+^T cell responses which also play a key role in protection (39). Recent studies on patients infected with SARS-CoV-2 have explained the important role of T-cell immune response in conferring protection (40). It will be interesting to study the anti-viral activities of these peptides specific to T cells.

These synthetic peptides are linear epitopes; hence the antibodies induced after immunizations might not be able to recognize conformational epitopes or glycan-occluded epitopes on the S protein. However, our results indicate that the immunized sera were able to bind to soluble S protein expressed in a mammalian system that is conformational, trimeric, fully glycosylated, and native-like (Figure 4) (41). Furthermore, the immunized sera were able to bind to denatured S protein and soluble RBD protein on SDS-PAGE as shown in the Western blot analysis. The results suggest that the epitopes are well-exposed to the S protein and flexible. Mapping of the epitopes on the S-protein structure (PDB: 6VYB) also suggests that the epitopes are well-exposed and easily accessible on the surface of the trimeric S protein. Neutralizing antibodies are reported for SARS-CoV that binds to the region corresponding to that of S1-pep1 in the N-terminal domain and RBD-Pep2 in RBD (23, 24). In this study, the highest humoral immune responses were directed toward S2-pep3 which is a part of HR2 domain. While neutralizing antibodies that bind to the region preceding immediately to the transmembrane region are reported for HIV envelope glycoprotein, a similar mechanism may be expected to block the infection of SARS-CoV-2 *via* S protein as it is also a class I fusion protein with a similar structural arrangement (42). Moreover, antibodies binding to the S protein at the membrane-proximal region may also interfere with the stability of the trimer or efficient formation of post-fusion conformation that is necessary for virus–host cell fusion process.

At lower dilutions of (1:20), the immunized sera from all the three peptides have shown to exhibit >50% neutralization against SARS-CoV-2 wild type USA-WA1/2020 isolate (Figure 5). In surrogate-based neutralization, the RBD-pep2 immunized sera further showed to inhibit the RBD-hACE2 interaction. The weak neutralization might be due to small linear epitopes and the usage of Freund's adjuvant. Nevertheless, further improvement in using multiple peptides or displaying these peptides in multiple arrays and using efficient adjuvant-like Addavax might increase the neutralizing responses. However, taken together the results suggest these peptides have the potential for the induction of both humoral and T-cell responses against SARS-CoV-2. These findings are important in developing peptide-based multiepitope or next-generation improved SARS-CoV-2 vaccines.

Anti-viral peptides are promising targets to act as viral entry inhibitors (43, 44) by interacting with the membrane surfaces or hydrophobic pockets and thus interfering in either virus attachment or fusion process. Xia et al. (36) have identified that lipopeptides targeted toward the HR1 domain of spike protein show high activity against virus fusion and thus efficiently inhibit the infection of the virus. In SARS, peptides derived from the HR motif of the S2 domain have been shown to be highly effective in inhibiting virus entry (45, 46). Here, the synthetic peptides, such as RBD-pep2 and S2-pep3 have shown to be inhibiting the virus entry in pre-fusion state at a concentration as low as ~2.5 μm as seen in CPE-based infectivity assay (Figure 5C). The RBD-pep2 is a part of RBM of RBD that interacts with the host receptor, ACE2 protein and thus might be playing a direct role by interfering in the viral attachment to the host cell. In the case of SARS coronaviruses, most of the characterized neutralizing antibodies partially or fully bind to the RBM region and block the accessibility of ACE2 (47). Our result with the monomeric peptide from the RBM suggests a promising possibility of designing a multimeric peptide-based or domain-based vaccine candidate that includes the RBM.

The S2-pep3 is present at the HR2 region of SARS-CoV-2 spike domain and found to be hydrophobic and rich in phenylalanine and glycine residues (Supplementary Figure 1). The heptad repeats HR1 and HR2 are responsible for the formation of a six-helix bundle, thus facilitating the fusion process. The S2-pep3 might be interfering in the fusion process, in the lipid membrane interface, thus inhibiting virus entry and replication (Figure 5D). Future studies on the mechanism of such interactions of the peptides within virus–host membrane pocket will further enable us in understanding the role of these peptides as anti-viral blocking agents.

In summary, the present study demonstrates the potential role of SARS-CoV-2 spike domain epitopes in the form of synthetic peptides to induce enhanced and desirable immunogenicity, which can be tailored in designing multimeric spike-based vaccine candidates. Multimeric homologous immunogens are adventitious in activating the B cell and are effective in cross-linking the B-cell receptor (BCR) (48, 49). In addition, the peptides also represent attractive targets to further exploit as “gatekeepers” or as “anti-viral inhibitors.”

## Materials and Methods

### Peptide Designing and Synthesis

The sequence of SARS-CoV-2 used for designing the peptides was based on the amino acid sequence of SARS-CoV-2 isolated Wuhan-Hu-1, GenBank: MN908947.3. Peptides of 20-mer were selected from three different regions of SARS-CoV-2 S protein, the NTD, the RBD, and the S2 domain. The peptide selected from NTD corresponds to a peptide of 236aa-253aa of SARS-CoV and was reported previously of very high antigenicity (23) and named as S1-pep1. The peptide selected from the RBD region is a part of the RBM; the corresponding region in SARS-CoV, a linear peptide of 460aa-476aa that binds to neutralizing antibodies F26G18 and F26G8 (24) and named as RBD-pep2. For the S2-domain, a 20-mer peptide was selected from the HR2 region of S2 domain (1186-1205 aa) and which was found to be highly antigenic in the antigenicity prediction tool and named as S2-pep3. For the B cell linear epitope prediction, Bepipred Linear Epitope Prediction 2.0 of the Immune Epitope Database and Analysis Recourse (iedb.org) was used. The three designed peptides were custom synthesized in conjugation with keyhole limpet hemocyanin (KLH) (GenScript, NJ, USA). The peptides were dissolved in the phosphate-buffered saline (PBS) at pH 7.4 to a final concentration of 1–4 mg/ml.

### Mouse Immunization

For immunization study, 7–8 weeks old female BALB/c mice weighing 18–25 gm and inbred in THSTI small animal facility (SAF) were used. Fifteen mice were randomly divided into five groups having three mice in each group (the smaller number of mice was used because of COVID-19 pandemic, and there was a partial lockdown of THSTI small animal facility). The animal study was conducted as per the institutional animal ethical regulations and Ethical Approval No. IAEC/THSTI/104. Mice were immunized with peptides using Freund's complete adjuvant for priming and Freund's incomplete adjuvant for boosting at 1:1 ratio (because of COVID-19 lockdown, we could not receive FDA approved adjuvant like AddaVax™). Control groups were administered PBS and the mice in Group 4 were given a mixture of peptides (S1-pep1, RBD-pep2, and S2-pep3) at equal concentration. Peptides were administered at a dose of 40 μg per mouse *via* intramuscular route (cranial thigh muscles) and similar doses were administered at each time point of 2-week intervals for all groups except the naïve group. The mice were observed daily and weighed twice a week over the course of the immunization period. Blood sample from each mouse was collected at day 0 (pre immune sera), 14 (sera after priming), 28 (sera after the first boost), and at 42 (sera after the second boost) days. Serum was separated from the blood, heat inactivated at 56°C for 1 h, and stored at −20°C for future use.

### Antigen Binding ELISA

His-tagged codon optimized genes of RBD and S protein for mammalian expression were used for the transient transfection of SARS-CoV-2 RBD and S protein ectodomain in Expi293F cells to produce the recombinant proteins and purified by Ni-NTA affinity chromatography following the standard protocol (27, 50). Maxisorp 96-well ELISA plates (Nunc, Thermo Scientific, Kamstrupvej, Denmark) were coated with 100 μl of 2 μg/ml concentration of respective peptides or RBD and S soluble protein overnight at 4°C in a coating buffer. To measure the binding of sera to conformational S protein, 1 μg/ml of the S trimeric protein was captured onto 96-well Ni-NTA HisSorb plates (Qiagen, MD, USA) in PBS overnight at 4°C. The wells were washed three times with PBS containing 0.05% phosphate-buffered saline with Tween-20 (PBST) and blocked with 5% skimmed milk for 1 h at room temperature. The mice antisera were diluted serially in a 2-fold series of PBS containing 0.5% skimmed milk to a final volume of 100 μl. The plate was incubated for 2 h at room temperature (RT) followed by washing three times with PBST. The bound primary antibody against each peptide was measured using HRP-conjugated anti-mouse IgG secondary antibody and its subclasses, such as IgG1, IgG2a, IgG2b, and IgG2c (Jackson Immunoresearch, PA, USA) (1:2000 dilution in PBS containing 0.5% skimmed milk). After incubation with secondary antibody at RT for 1 h, the plates were washed four times with PBST and developed using 100 μl of tetramethylbenzidine (TMB) substrate (Thermo Fisher Scientific, MA, USA). The reaction was stopped by adding 2N H_2_SO_4_ and absorbance was measured at 450 nm. The endpoint titer was calculated as the reciprocal serum dilution giving O.D 450 nm readings >2 and the background levels which was calculated using pre-bleed serum at the same dilutions.

### Western Blot Analysis

For Western blot analysis, the S and RBD soluble proteins were separated in 12% sodium dodecyl sulfate-polyacrylamide (SDS-PAGE) gel and transferred to a polyvinylidene fluoride (PVDF) membrane. The membrane was blocked with 5% skimmed milk, incubated with pooled sera from S1-pep1 (1:100), RBD-pep 2 (1:1000), and S2-pep 3 (1:100) immunized group for overnight at 4°C. The membrane was developed with HRP-conjugated anti-mouse secondary antibody (Jackson ImmunoResearch, PA, USA).

### Binding of Antibodies to SARS-CoV-2 Prefusion-Spike Protein

For measuring the binding of antibodies to the spike protein stabilized in pre-fusion conformation (the construct is S1–S2 cleavage deficient with 2P mutations in the HR1 region) (NR-52394, BEI Resources, NIAID, NIH) (6), anti-mouse Fc sensors (ForteBio Inc., Ca. USA) was used to capture the mouse IgGs purified from the immunized mice sera using protein A affinity purification. The soluble spike protein in pre-fusion conformation was used as an analyte at a concentration of 1 um in the PBS buffer background supplemented with 0.1% bovine serum albumin (BSA) and 0.02% Tween. The ligands were used at a concentration of 10 μg/ml. Associations and dissociations were recorded for 600 s. The experiment was performed at RT with agitation at 1,000 rpm. The data were analyzed using the ForteBio Data Analysis software, 10.0 (Forte-Bio Inc., CA, USA).

### Confocal Immunofluorescence Analysis

Vero E6 cells were grown in coverslip bottom dishes of diameter, 35 mm (Biogenuix, New Delhi, India) and infected with SARS-CoV 2, Isolate USA-WA1/2020 virus using a multiplicity of infection (MOI) of 10. After 24-h post-infection (pi), the cells were fixed with 100% chilled methanol for 20 min in ice. In order to visualize the infected cells intracellularly by immunofluorescence, fixed cells were blocked with 3% goat sera for 1 h at RT followed by incubation with pooled sera (1:50 dilution of S1-pep1 and S2-pep3 and 1:100 dilution of RBD-pep2 pooled sera) from each immunized group and PBS-control sera (1:100 dilution) for 3 h at RT. The cells were then washed in PBS three times and stained with the anti-mouse AlexaFluor 488 secondary antibody (Invitrogen Corporation, CA, USA) at 1:1000 for 1 h at RT. The cells were mounted using ProLong Gold anti-fade reagent with DAPI (Invitrogen Corporation, CA, USA). Images were acquired on an Olympus FV3000 confocal microscope with 60X (NA 1.4) Plan Apo objectives.

### *In vitro* Stimulation of Splenocytes

The spleen from the immunized mice was isolated and an RBC lysed single-cell suspension was prepared. About 0.5 million splenocytes were then cultured in 96-well plates in Iscove's Modified Dulbecco's Medium (IMDM) complete media and stimulated with (a) PMA (20 ng/ml) and ionomycin (1 μg/ml) for 4 h or (b) stimulated in the presence of 15 μg of S1-pep1, RBD-pep2, S2-pep3 peptide, or mixed (cocktail)-prep (containing 5 μg of each peptide) for 5 h in 5% CO2 incubator at 37°C. Thereafter, a culture soup was collected for ELISA and the cells were washed and staining was carried out for intracellular cytokines. Immunophenotyping was done from the spleen of three different animals of the same group.

Intracellular cytokine staining: *in vitro*-stimulated splenocytes were first surface stained for CD45.2, CD4, and CD8 markers at RT for 20 min in dark and thereafter fixed and permeabilized with BD Cytofix/Cytoperm (BD Biosciences, CA, USA) according to the manual of the manufacturer. The permeabilized cells were then stained for intracellular cytokines, such as IFNγ, interleukin (IL)-2, and IL-17 at RT for 20 min in dark. The cells were then washed and acquired on BD FACSCanto II (BD Biosciences, CA, USA). The acquired samples were then analyzed on FlowJo software (Tree Star, Inc., OR, USA) and plotted on GraphPad prism software.

### ACE2-RBD Competition ELISA

For ACE2-RBD competition assay, Fc-conjugated soluble ACE2 (ACE2-Fc plasmid is a kind gift from Prof. S Pöhlmann, Infection Biology Unit, Göttingen, Germany) was coated with the ELISA plate (100 μl/well of 2 μg/ml) overnight. Next day, the plate was washed and blocked with 2% skimmed milk for 1 h. A 100 μl mix containing 100 ng of soluble RBD-His and different dilutions of mice sera from immunized mice or peptide with different concentrations were pre-incubated for 1 h or 30 min, respectively at RT. Different dilutions of mice pooled sera from S2-pep3 or peptide dilution was used as a negative control. The mix was then transferred to ACE2-Fc coated ELISA plate after removing milk and washed one time with PBST and further incubated for 1 h at RT. The plate was then washed and HRP-conjugated anti-His antibody was added for 1 h and incubated at RT; the plate was washed two times and then developed with TMB substrate. The reaction was stopped by adding 2N H_2_SO_4_ and the absorbance was measured at 450 nm.

### SARS-CoV-2 Wild-Type Virus Neutralization Assay

SARS-Related Coronavirus 2, Isolate USA-WA1/2020 virus was used to perform plaque-based neutralization assay in THSTI Infectious Disease Research Facility (Biosafety level 3 facility). Briefly, 100 μl of pooled sera from immunized groups were incubated with 100 μl of 50 PFU of SARS-CoV-2 Isolate USA-WA1/2020 virus at 1:10 and 1:20 dilution for 1 h at 37°C. Following incubation, confluent Vero E6 cells in 24-well plates (seeded onto the 24-well tissue culture plates, the day before infection) were infected with the sera-virus mixture for 1 h at 37°C after removing the growth medium. After 1 h of adsorption, the plate was washed one time with Dulbecco's Modified Eagle Medium (DMEM) growth media and overlaid with DMEM growth medium containing 2% carboxy methylcellulose (CMC) and incubated at 37°C. Forty-eight hours p.i., the overlay was removed and the cells were washed with PBS and fixed in 6% formalin for inactivation of virus for 4 h at RT. The fixed-cell monolayers were stained with 1% crystal violet for 30 min at RT and washed under running tap water. The plates were allowed to air dry, and the plaques were visualized and counted by eye.

### Peptide SARS-CoV-2 Wild-Type Inhibition Assay

Wild-type SARS-CoV-2 Isolate USA-WA1/2020 viruses (50 μl of 10 MOI) were co-incubated with 50 μl peptides of different concentrations at 37°C for 30 min. The mixture was then transferred to Vero E6 cells seeded in 24-well plates and incubated for 1 h at 37°C. After incubation, the medium in each well was then replaced with 200 μl of 2% DMEM and incubated at 37°C in the CO_2_ incubator. To measure the effectiveness of the peptide after the virus attachment, the untreated virus was allowed to attach for 1 h at 37°C, followed by washing with PBS and then added with 2% DMEM containing 2.5–5 μm peptide and incubated at 37°C in the CO_2_ incubator. Forty hours p.i., the cytopathic effect in each well was visualized in a microscope.

### Structural Modeling

The regions of the SARS-CoV-2 spike protein that were selected for designing the peptides have missing electron density on the available structures of the ectodomain of the S protein. To model the missing region from the S1-pep1 and RBD-pep2 peptides, we used a SWISS MODEL using PDB; 6VYB as a template. The model structure is superimposed with the template structure (6VYB) with an RMSD of 0.47. To build a model for the C terminal region of S2, the S2 amino acid sequence was used in int-FOLD server, the best model superimposed with the template, 6VYB, with an RMSD of 0.95. This model structure was used to view the location of the selected peptide regions.

### Statistics

Statistical analyses were performed using the analysis software within the GraphPad Prism package 8. For ELISA, we analyzed our data using one-way ANOVA and Tukey's multiple comparisons test to understand if there is an interaction between each animal sera as an independent variable on the dependent variable which is the coated protein, and statistical significance was determined by utilizing the analysis of variance taking into consideration, the variation of all data from experimental groups, where *p* < 0.05 was considered significant. The T-cell assays were analyzed using one-way Anova, where ^*^p < 0.05, ^**^p < 0.01, ^***^p < 0.001, and ^****^p < 0.0001 were considered significant.

## Data Availability Statement

The original contributions presented in the study are included in the article/Supplementary Material, further inquiries can be directed to the corresponding authors.

## Ethics Statement

The animal study was reviewed and approved by Institutional Animal Ethics Committee, THSTI.

## Author Contributions

PV and NY carried out animal experiments. SS, SA, PV, NY, NK, AC, and MB carried out biochemical and biophysical experiments. SS and PD carried out BSL3 virus experiments. AA and ZR executed cytokine expression experiments and analyzed the data. TS carried out the soluble RBD and spike protein production. RK edited the manuscript. SS and SA conceived the study, designed experiments, performed experiments, and analyzed data. SS wrote the original draft. SA edited the manuscript. All authors contributed to the article and approved the submitted version.

## Conflict of Interest

The authors declare that the research was conducted in the absence of any commercial or financial relationships that could be construed as a potential conflict of interest.
