# Supplementary material for: Severe Acute Respiratory Syndrome Coronavirus 2 Spike Protein Based Novel Epitopes Induce Potent Immune Responses in vivo and Inhibit Viral Replication in vitro
Source: Front Immunol. 2021 Mar 26;12:613045. doi: 10.3389/fimmu.2021.613045 (PMC8032902; doi:10.3389/fimmu.2021.613045)
Supplement: Supplementary file 1 [file Data_Sheet_1.docx]

**Supplementary Material**

**Severe Acute Respiratory Syndrome Coronavirus 2 Spike Protein Based Novel Epitopes Induce Potent Immune Responses in vivo and Inhibit Viral Replication in vitro**

Preeti Vishwakarma†, Naveen Yadav †, Zaigham Abbas Rizvi, Naseem Ahmed Khan, Adarsh Kumar Chiranjivi, Shailendra Mani, Manish Bansal, Prabhanjan Dwivedi, Tripti Shrivastava, Rajesh Kumar, Amit Awasthi, Shubbir Ahmed* and Sweety Samal*

Translational Health Science & Technology Institute, National Capital Region (NCR) Biotech Science Cluster, Faridabad, India

**Abstract**

Severe acute respiratory syndrome coronavirus 2 (SARS-CoV-2) initiates infection by attachment of the surface-exposed spike glycoprotein to the host cell receptors. The spike glycoprotein (S) is a promising target for inducing immune responses and providing protection; thus the ongoing efforts for the SARS-CoV-2 vaccine and therapeutic developments are mostly spiraling around S glycoprotein. The matured functional spike glycoprotein is presented on the virion surface as trimers, which contain two subunits, such as S1 (virus attachment) and S2 (virus fusion). The S1 subunit harbors the N-terminal domain (NTD) and the receptor-binding domain (RBD). The RBD is responsible for binding to host-cellular receptor angiotensin-converting enzyme 2 (ACE2). The NTD and RBD of S1, and the S2 of S glycoprotein are the major structural moieties to design and develop spike-based vaccine candidates and therapeutics. Here, we have identified three novel epitopes (20-amino acid peptides) in the regions NTD, RBD, and S2 domains, respectively, by structural and immunoinformatic analysis. We have shown as a proof of principle in the murine model, the potential role of these novel epitopes in-inducing humoral and cellular immune responses. Further analysis has shown that RBD and S2 directed epitopes were able to efficiently inhibit the replication of SARS-CoV-2 wild-type virus in vitro suggesting their role as virus entry inhibitors. Structural analysis revealed that S2-epitope is a part of the heptad repeat 2 (HR2) domain which might have plausible inhibitory effects on virus fusion. Taken together, this study discovered novel epitopes that might have important implications in the development of potential SARS-CoV-2 spike-based vaccine and therapeutics.

**Keywords: spike (S) glycoprotein, peptide, vaccine, antiviral activity, immune response**

**Table of contents**

**Supplementary Figure 1.** Linear epitope prediction of SARS-CoV2 spike protein

**Supplementary Figure 2:** IgG1, IgG2a,2b and IgG2c antibody levels as measured by ELISA after second boost to synthetic peptides

**Supplementary Figure 3:** Intracellular cytokine staining for splenocytes stimulated by PMA + Ionomycin in vitro.

**Supplementary Figure 4:** High resolution picture of plaque neutralization and CPE assay as shown in figure 5

**Supplementary figure 1:** **Linear epitope prediction of SARS-CoV-2 spike protein**


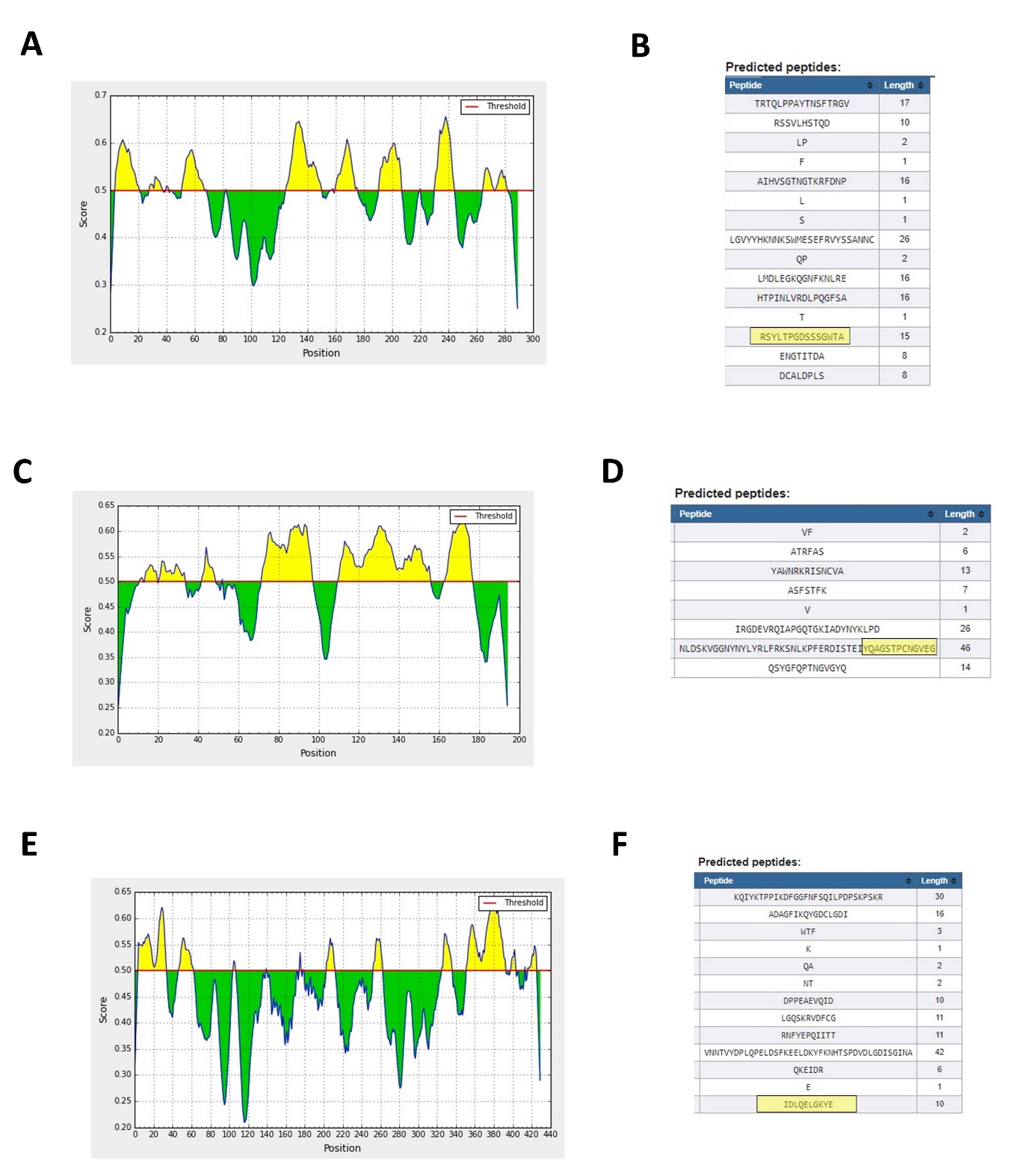


**Supplementary figure 1**. Linear epitope prediction. In the right panel the yellow peaks correspond to the linear epitopes and in the left panel shows the sequence of predicted peptides and their length. The boxed region includes the sequence, partly or fully, of the peptides used in this study. A) and B) is the data for the NTD region (S1-pep1), C) and D) is the data for RBD region E) and F) is the data from the S2 region of spike protein.

**Supplementary figure 2.**


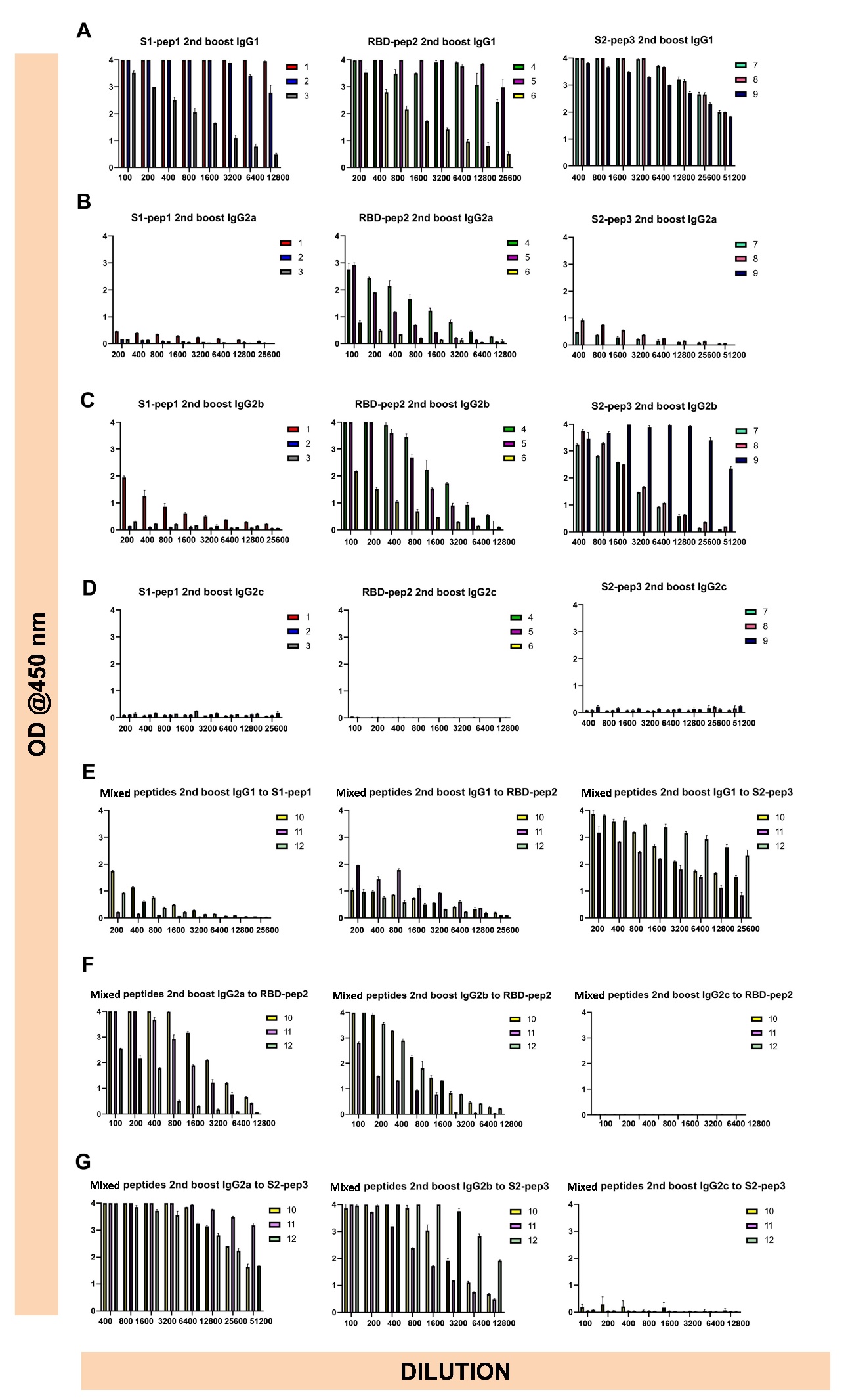


**Supplementary figure 2. IgG1, IgG2a,2b and IgG2c antibody levels as measured by ELISA after second boost to synthetic peptides.** A. IgG1 antibody titers B. IgG2a antibody titers C. IgG2b antibody titers of immunized sera to S1-pep1 (left lane), RBD-pep2 (middle lane) and S2-pep3 (right lane) respectively. D. IgG2c antibody titers of immunized sera to S1-pep1 (left lane), RBD-pep2 (middle lane) and S2-pep3 (right lane) respectively E. IgG1 antibody titer response of mixed peptides immunized sera to S1-pep1 (left lane), RBD-pep2 (middle lane), and S2-pep3 (right lane). F. IgG2a (left lane), IgG2b (middle lane) and IgG2c (right lane) antibody titers of mixed peptides immunized sera to RBD-pep2 G. IgG2a (left lane), IgG2b (middle lane) and IgG2c (right lane) antibody titers of mixed peptides immunized sera to S2-pep3.

**Supplementary Figure 3.**


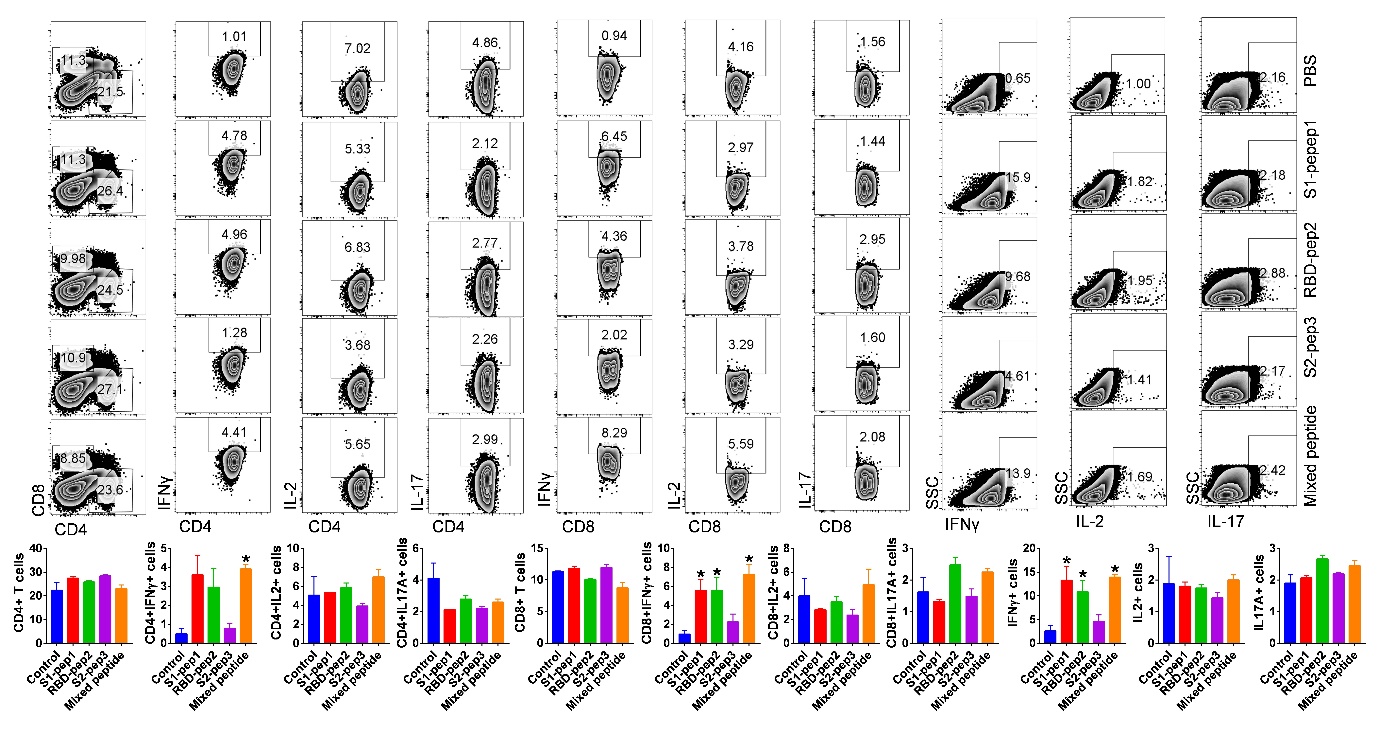


**Supplementary Figure 3:** **Intracellular cytokine staining for splenocytes stimulated by PMA+Ionomycin *in vitro*.** PMA+Ionomycin stimulated splenocytes from immunized animals were stained for IFNγ, IL2 and IL17 cytokines after CD4 and CD8 surface staining. Stained cells were then acquired on BD FACSCanto II and analyzed on FlowJo. A.Top panel shows representative dot plots indicating mean % positive values for various T cell populations in stimulated spleen. B.Bar graph plotted for percent positive ± standard errors of the mean (SEMs) for each group are shown (Bottom). *P < 0.05 (One-way anova).

**Supplementary Figure 4A.**


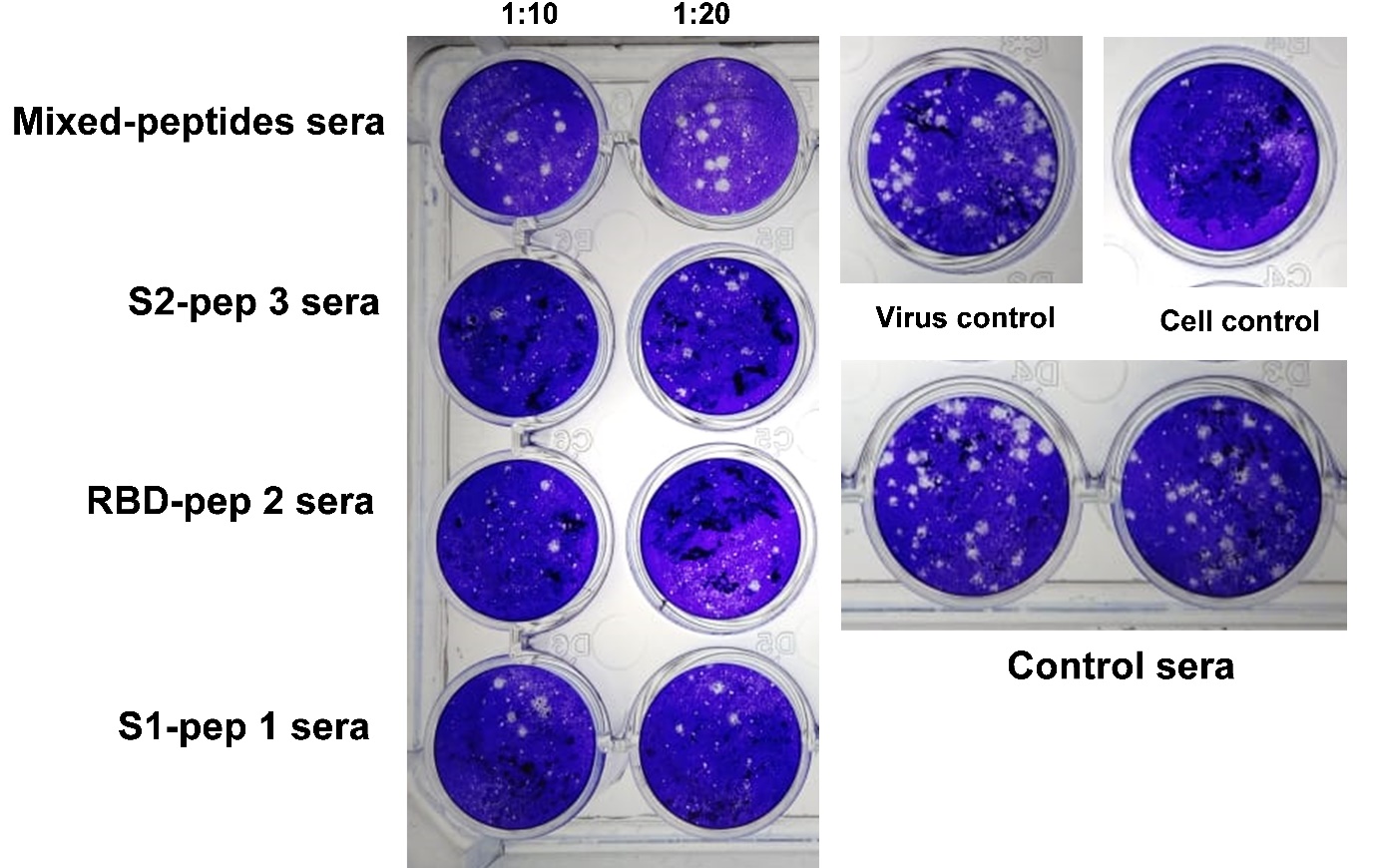


**Supplementary figure 4A:** High resolution pictures of Figure 5A from main manuscript for SARS-CoV-2 wildtype neutralization assay.

**Supplementary Figure 4B**


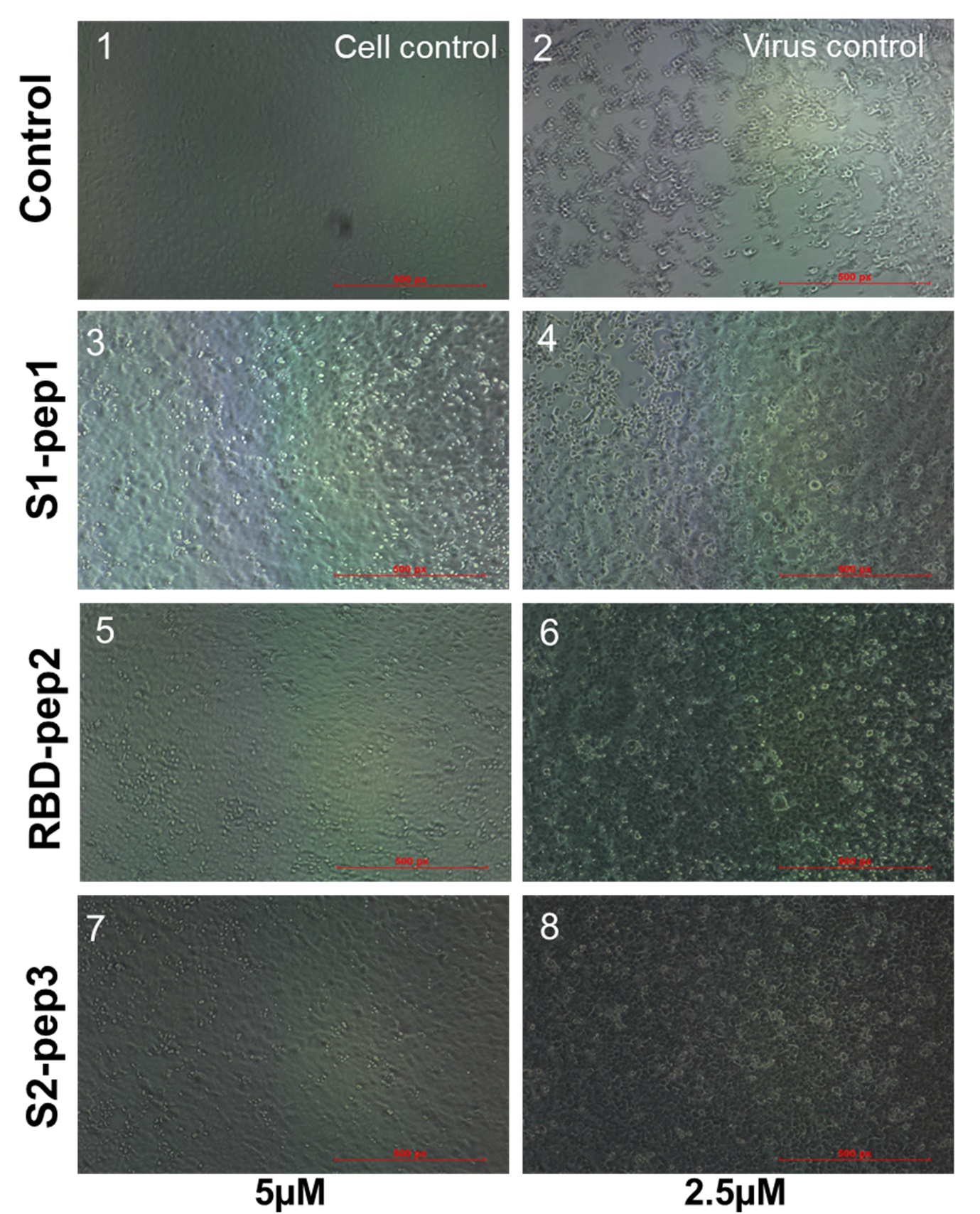


**Supplementary figure 4B:** High resolution picture of Figure 5C from main manuscript for

Cytopathic effect (CPE) based viral inhibition assay with peptides.

**Supplementary figure 4C**


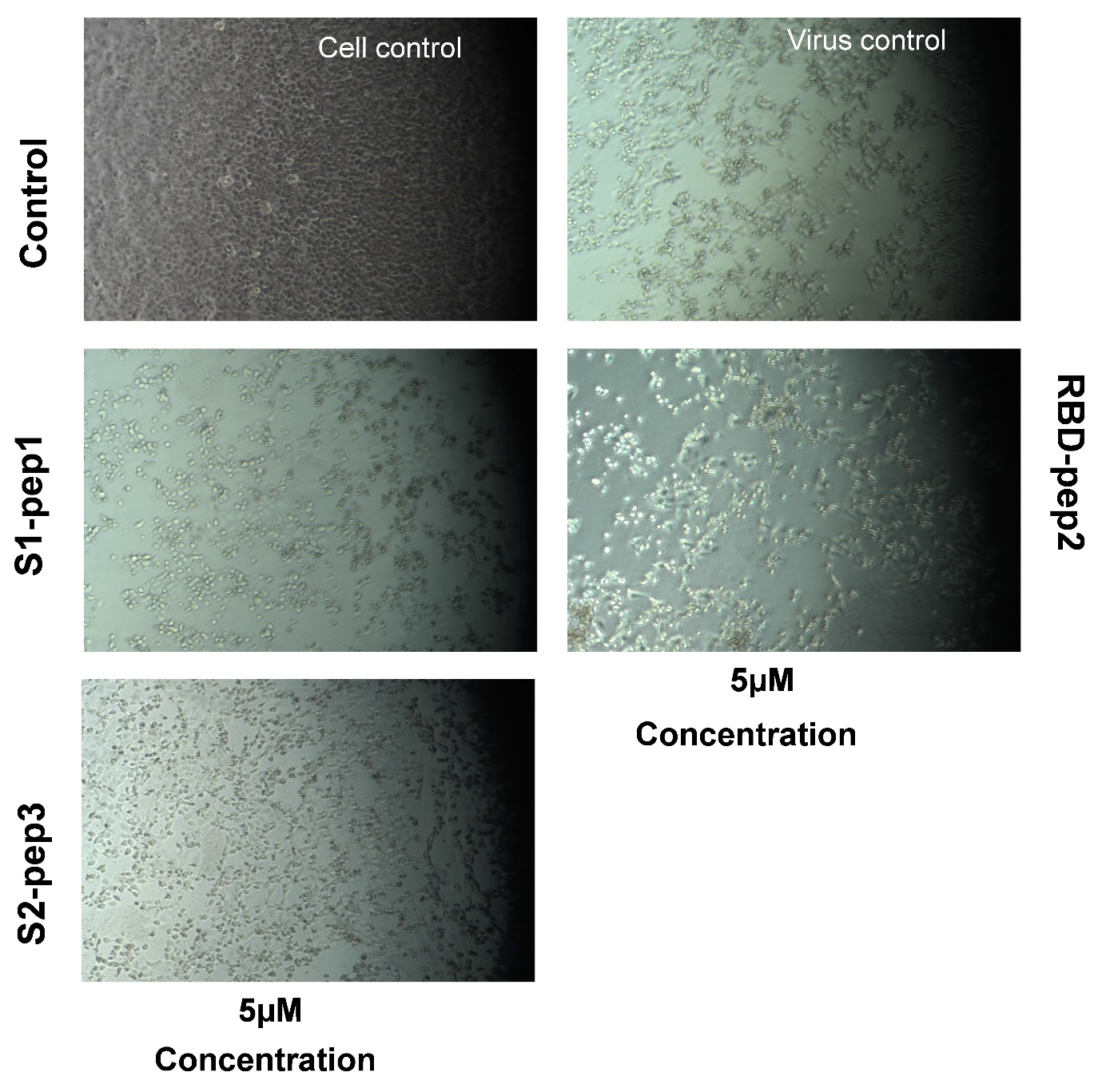


**Supplementary figure 4C:** High resolution picture of Figure 5D from main manuscript for

Cytopathic effect (CPE) based viral inhibition assay with peptides after virus attachment.
